# Effect of Ni Core Structure on the Electrocatalytic Activity of Pt-Ni/C in Methanol Oxidation

**DOI:** 10.3390/ma6072689

**Published:** 2013-07-08

**Authors:** Jian Kang, Rongfang Wang, Hui Wang, Shijun Liao, Julian Key, Vladimir Linkov, Shan Ji

**Affiliations:** 1 Key Laboratory of Eco-Environment-Related Polymer Materials, Ministry of Education of China, College of Chemistry and Chemical Engineering, Northwest Normal University, Lanzhou 730070, China; E-Mails: kangjian_2006@126.com (J.K.); wanghui3931@126.com (H.W.); 2 Key Laboratory of Fuel Cell Technology of Guangdong Province, School of Chemistry and Chemical Engineering, South China University of Technology, Guangdong, Guangzhou 510640, China; E-Mail: chsjliao@scut.edu.cn; 3 South African Institute for Advanced Materials Chemistry, University of the Western Cape, Cape Town 7535, South Africa; E-Mails: joolskey@uwc.ac.za (J.K.); vlinkov@uwc.ac.za (V.L.)

**Keywords:** electrocatalysts, amorphous, core-shell structure, fuel cell, methanol oxidation

## Abstract

Methanol oxidation catalysts comprising an outer Pt-shell with an inner Ni-core supported on carbon, (Pt-Ni/C), were prepared with either crystalline or amorphous Ni core structures. Structural comparisons of the two forms of catalyst were made using transmission electron microscopy (TEM), X-ray diffraction (XRD) and X-ray photoelectron spectroscopy (XPS), and methanol oxidation activity compared using CV and chronoamperometry (CA). While both the amorphous Ni core and crystalline Ni core structures were covered by similar Pt shell thickness and structure, the Pt-Ni(amorphous)/C catalyst had higher methanol oxidation activity. The amorphous Ni core thus offers improved Pt usage efficiency in direct methanol fuel cells.

## 1. Introduction

Increasing Methanol oxidation in direct methanol fuel cells affords an important alternative fuel source for future energy requirements [1]. In the search for efficient electrocatalysts that offset CO poisoning of the Pt-based anode, binary [2,3] or ternary [4,5] metallic systems in the form of alloys or core-shell structures have shown considerable promise for methanol oxidation. Among these catalysts, the PtNi bimetallic alloy has received much attention [6,7]. It was reported that the rate of methanol oxidation is depended on factors such as: the amount of second metal content, the degree of its alloying with Pt, electrode pretreatment, and the type of supporting substrate [8,9,10,11]. To form core-shell PtNi structures is an efficient way to improve the performance, not only in the activity of catalyst but also in the utilization of Pt [12,13,14]. The Sotiropoulos group found that a Ni core with a Pt shell nanocatalyst resulted in moderate improvement of methanol oxidation activity during medium-term experiments [15]. Moreover, the Li group reported that the porous Ni@Pt core-shell nanotube arrays showed markedly enhanced methanol oxidation activity and stability compared to commercial Pt/C catalysts [16]. However, the above-mentioned studies did not investigate the effect of thickness of the shell and core structure on catalytic activity.

Pt shell thickness in the Pt shell-core structure has been found to effect methanol oxidation activity in other catalysts [17,18]. The Sung group examined Ir core size, finding that smaller core sizes produced greater catalytic activity than larger [19]. Also, the use of an amorphous structured metal core, compared to a crystalline core, improved catalytic activity in a Fe-core/Pt-shell catalyst for ammonia borane oxidation [20]. These reports thus indicate that both the thickness of shell, and the size and crystallinity of the core, can influence catalyst activity. However, reports on factors effecting core-shell catalyst activity are far fewer than those on factors effecting alloys such as, the synthesis method, the metal ratio and the alloying degree.

In the present study, catalysts containing an outer Pt shell with an inner Ni core supported on carbon were prepared with either amorphous or crystalline Ni cores (denoted as Pt-Ni_A_/C and Pt-Ni_C_/C respectively) through modification using a two-step method. Pt-Ni_A_/C was found to be more active toward methanol oxidation catalyst than Pt-Ni_C_/C.

## 2. Results and Discussion

**Figure 1:**
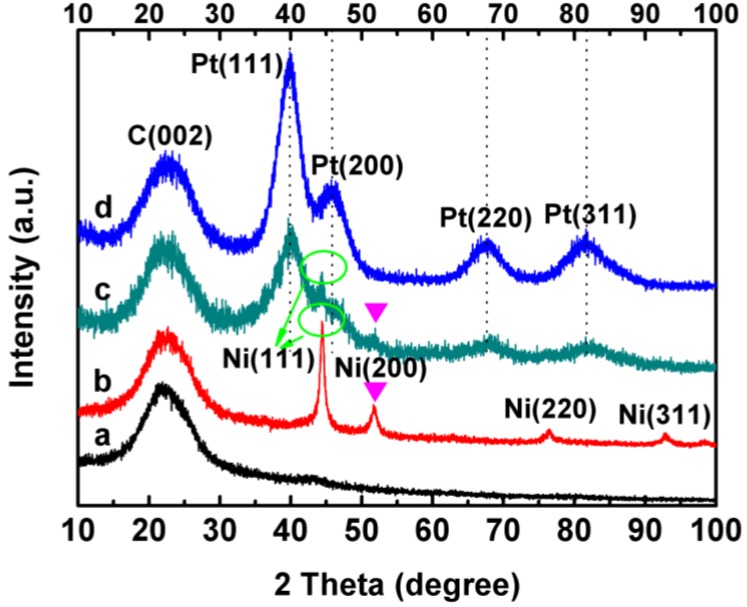
X-ray diffraction patterns of (**a**) Ni_A_/C; (**b**) Ni_C_/C; (**c**) Pt-Ni_C_/C and (**d**) Pt-Ni_A_/C; the vertical dot lines correspondent to the positions of diffraction peaks for the home-made Pt/C sample.

Figure 1 shows the XRD patterns of Ni_A_/C, Ni_C_/C, Pt-Ni_C_/C and Pt-Ni_A_/C nanoparticles. For comparison, the XRD pattern of a home-made Pt/C sample is also displayed as the vertical dot line. The first peak located at ~24.8° in all the XRD patterns is associated with the carbon support. For Ni_A_/C (pattern a, Figure 1) the slight peak at 2*θ* values of 42°~48° suggests that the Ni is amorphous [21]. For the Ni_C_/C (pattern b, Figure 1), all of the peaks match well with Bragg reflections of the standard face-centered cubic (fcc) structure (ICDD file No.: 04-0850, space group: Fm3m (225)), the four peaks at 2*θ* = 44.5°, 51.8°, 76.3° and 92.8° can be assigned to their characteristic (111), (200), (220) and (311) indices [22,23].

After depositing Pt on Ni nanoparticles via a reduction reaction, both Pt-Ni_C_/C (pattern c, Figure 1) and Pt-Ni_A_/C (pattern d, Figure 1) had diffraction peaks at 39.8°, 45.8°, 67.4° and 81.5°, which can be assigned to (111), (200), (220) and (311) planes of the Pt-shell, suggesting that the structure of Pt layers was not affected by the structural difference of Ni nanoparticles. For Pt-Ni_C_/C (pattern c, Figure 1), well separated diffraction peaks suggest the presence of different phases. Diffraction peaks at 44.5° and 51.8° agreed with a metallic Ni structure assigned to the Ni-core. In contrast, the XRD pattern for Pt-Ni_A_/C (pattern d, Figure 1) lacked the corresponding Ni diffraction peaks. In addition, the diffraction angle phases of the shell and core for Pt-Ni_C_/C and Pt-Ni_A_/C did not shift to obviously higher angles or lower angles than those for Pt/C (see the dot line) and Ni/C, indicating that no alloy phase was formed.

**Figure 2:**
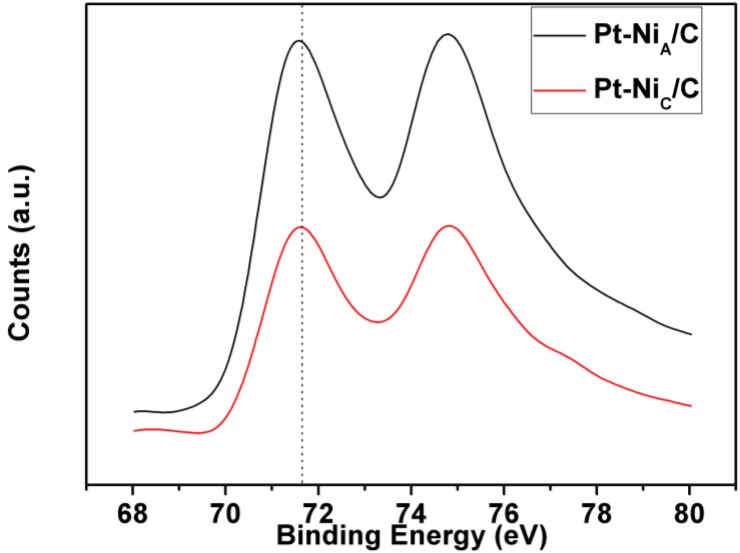
Pt 4f X-ray photoelectron spectroscopy (XPS) spectra of the Pt-Ni_C_/C and Pt-Ni_A_/C catalysts.

X-ray photoelectron spectroscopy (XPS) technique is a useful tool to study changes in the electronic structure of different surface preparations and compositions [24]. The regions of Pt 4f in the XPS spectrum of Pt-Ni_C_/C and Pt-Ni_A_/C are shown in Figure 2. The energy separation between Pt 4f_7/2_ and 4f_5/2_ is *ca.* 3.3 eV, which also adheres to the reference value [25]. Compared with the binding energy of Pt 4f of Pt-Ni_C_/C, that of Pt-Ni_A_/C shifts to the lower binding energy, which is likely contributed from lattice strain on the Pt shell and charge transfer between Ni atoms and Pt atoms [26]. The above result thus shows that the Pt-Ni_C_/C and Pt-Ni_A_/C had different electronic structure.

**Figure 3:**
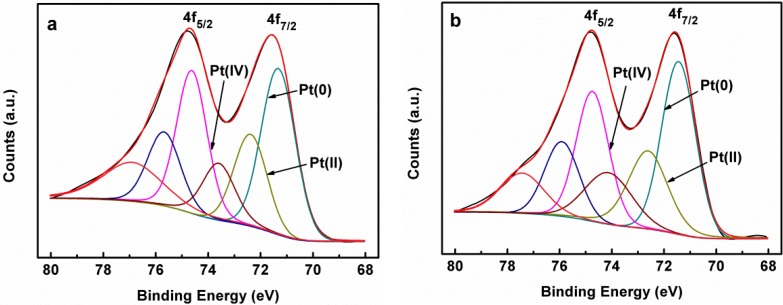
Lorentzian curves of Pt 4f XPS spectra of the Pt-Ni_A_/C (**a**) and Pt-Ni_C_/C (**b**) catalysts respectively.

In order to evaluate the composition of Pt species, the spectra were fitted as two pairs of overlapping Lorentzian curves. Figure 3 shows Lorentzian curves of the Pt 4f region spectrum of Pt-Ni_C_/C and Pt-Ni_A_/C, which could be deconvoluted into three pairs of doublets. In the Figure 3a, the most intense doublet (at 71.3 and 74.6 eV) listed in Table 1 is the signature of metallic Pt, and the theoretical ratio of peak areas is 4:3 for the Pt 4f_7/2_ and 4f_5/2_ lines respectively. The second and weaker doublet (at 72.4 and 75.7 eV), with binding energy at 1.1 eV higher than Pt(0), could be assigned to the Pt(II) oxidation state species in PtO and Pt(OH)_2_ [27,28]. The third doublet, which is the weakest in intensity and at even higher binding energy (at 73.6 and 76.9 eV), was most likely caused by a small amount of Pt(IV) residue on the surface [29]. For Pt-Ni_C_/C, the peak positions of the Pt 4f correspondent to the different valence were listed in Table 1. A comparison of the relative areas (Table 1) of integrated intensity of Pt(0), Pt(II), and Pt(IV) showed that the Pt on the surface of Pt-Ni_A_/C was predominately metallic (53.0%). The Pt 4f_7/2_ spectrum (Figure 3a) of Pt-Ni_C_/C also identified the presence of Pt(0), Pt(II) and Pt(IV), and the Pt on the surface of Pt-Ni_C_/C catalyst was also predominately metallic.

**Figure 4:**
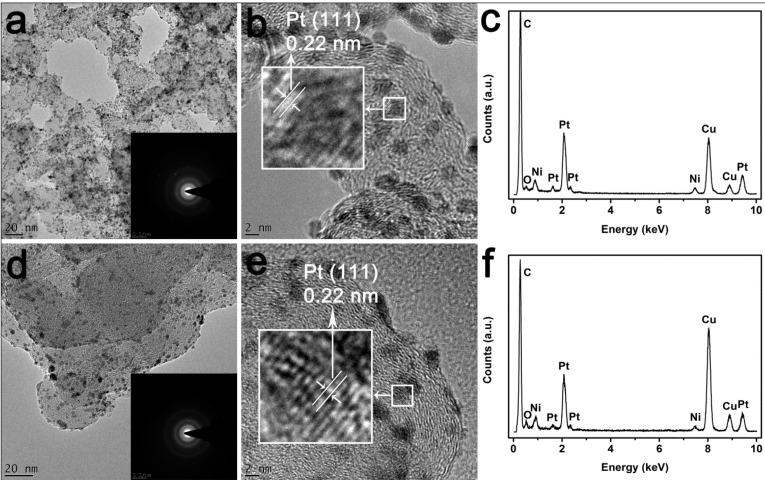
Transmission electron microscopy (TEM), high-resolution TEM (HRTEM) images and EDS spectrum of Pt-Ni_C_/C (**a**–**c**) and Pt-Ni_A_/C (**d**–**f**) catalysts, respectively. Inset of (**a**) and (**d**) show the selected area electron diffraction (SAED) patterns of Pt-Ni_C_/C and Pt-Ni_A_/C catalysts.

Figure 4 shows representative transmission electron microscopy (TEM), high-resolution TEM (HRTEM) images and EDS spectrum of Pt-Ni_C_/C and Pt-Ni_A_/C respectively. It can be seen that the Pt-Ni_C_ and Pt-Ni_A_ nanoparticles were highly dispersed on the carbon support with narrow size distribution. By counting one hundred particles, the average particle size of Pt-Ni_C_/C and Pt-Ni_A_/C was approximately 3.2 nm and 3.4 nm respectively. The selected-area electron-diffraction (SAED) pattern (inset of Figure 4a,d) of Pt-Ni_C_/C and Pt-Ni_A_/C catalysts showed a ring-like pattern with many spots, revealing that they indexed to planes of polycrystalline Pt. The similar SAED results for Pt-Ni_C_/C and Pt-Ni_A_/C again further suggests that the structure of Pt shell was similar for the two catalysts.

**Table 1:** Chemical States, binding energy, and area ratio of Pt-Ni_C_/C and Pt-Ni_A_/Ccatalysts by XPS.

Samples	Items	Pt 4f_7/2_		
		Pt(0)	Pt(II)	Pt(IV)
Pt-Ni_C_/C	Binding energy (eV)	71.4	72.5	74.1
	Area ratio (%)	49.6	29.2	21.2
Pt-Ni_A_/C	Binding energy (eV)	71.3	72.4	73.6
	Area ratio (%)	53.0	28.0	19.0

Figure 4b,e shows the HRTEM images of Pt-Ni_C_/C and Pt-Ni_A_/C. The measured distance between the two nearest atom rows for Pt-Ni_C_/C and Pt-Ni_A_/C was *ca.* 0.22 nm, which is close to the (111) interplanar distance of pure Pt (0.23 nm), suggesting a Pt shell formed on the Ni nanoparticles. At the same time, the reduction in the interplanar distance of Pt in Pt-Ni_C_/C and Pt-Ni_A_/C, compared to pure Pt, most likely results from the strain effect on the Pt shell from the Ni core. From the EDS spectrum, Figure 4c,f, the presence of C, Pt, and Ni elements were confirmed, and the atomic ratio of Pt/Ni of Pt-Ni_C_/C was 1:1.92, similar to that of Pt-Ni_A_/C (1:1.93), the metal loading for the two catalysts is *ca.* 35%, which was also confirmed by ICP analysis.

**Figure 5:**
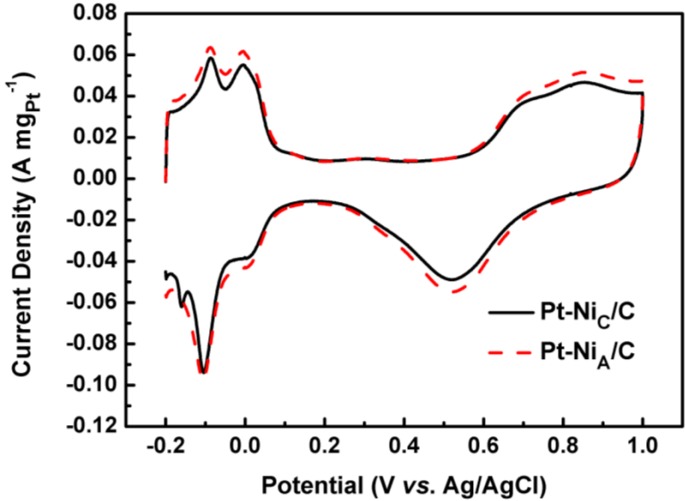
Cyclic voltammograms of Pt-Ni_A_/C and Pt-Ni_C_/C catalysts in 0.5 mol L^−1^ H_2_SO_4_ solution under N_2_ atmosphere at room temperature; Scan rate: 50 mV s^−1^.

Figure 5 shows cyclic voltammograms for Pt-Ni_A_/C and Pt-Ni_C_/C after stabilization (20 cycles) in a N_2_-saturated 0.5 mol L^−1^ H_2_SO_4_ solution at a scan rate of 50 mV s^−1^. There are three characteristic potential regions of polycrystalline Pt on both Pt-Ni_A_/C and Pt-Ni_C_/C: the hydrogen adsorption and desorption region (−0.2 to 0.1 V *vs.* Ag/AgCl), double layer plateau region (0.1~0.5 V *vs.* Ag/AgCl), and the formation and reduction of surface metals oxide (0.5~1.0 V *vs.* Ag/AgCl). The coulombic charge for hydrogen adsorption (*Q_H_*) is used to evaluate the electrochemical surface area (*S_ESA_*) of the electrocatalysts using Equation (1) [30], in which *m* represents the platinum loading (g) on the electrode, *Q_H_* is the charge for hydrogen desorption electro-oxidation in microcoulomb (C), and *C* is the charge required to oxidize a monolayer of H_2_ on the catalyst, its value is 0.21 mC cm^−2^.

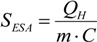
(1)
*S_ESA_* of the Pt-Ni_A_/C and Pt-Ni_C_/C were 106.1 and 89.5 m^2^ g_Pt_^−1^ respectively. The larger *S_ESA_* of the Pt-Ni_A_/C was slightly larger than that of the Pt-Ni_C_/C catalyst.

**Table 2:** Forward peak current density (*I_f_*), and reverse peak current density (*I_b_*), for ethanol oxidation on different Pt-based catalysts recorded in 0.5 mol L^−1^ H_2_SO_4_ + 0.5 mol L^−1^ CH_3_OH solution.

Samples	*I_f_* (A mg_Pt_^−1^)	*I_b_*(A mg_Pt_^−1^)
Pt-Ni_A_/C	0.378	0.448
Pt-Ni_C_/C	0.334	0.435

**Figure 6:**
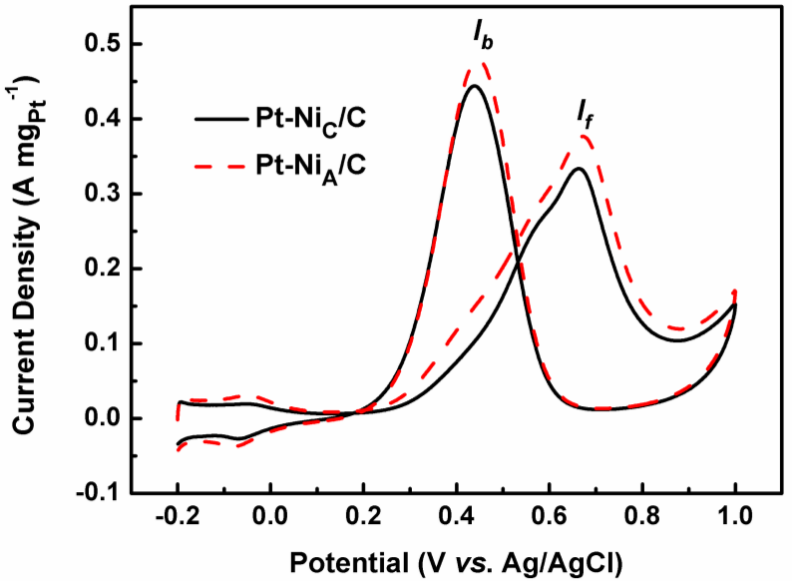
Cyclic voltammograms of the Pt-Ni_A_/C and Pt-Ni_C_/C catalysts in 0.5 mol L^−1^ H_2_SO_4_ + 0.5 mol L^−1^ CH_3_OH solution saturated by N_2_ at room temperature with scan rate of 50 mV s^−1^.

CVs were used to characterize catalytic activity in 0.5 mol L^−1^ H_2_SO_4_ + 0.5 mol L^−1^ CH_3_OH solution (Figure 6). Typical features of methanol oxidation were observed: two oxidation peaks, corresponding to the oxidation of methanol and intermediates, which occurred at *ca.* 0.68 and 0.46 V, respectively. The peak current densities are listed in Table 2 showing that Pt-Ni_C_/C < Pt-Ni_A_/C. The onset potential of methanol oxidation on Pt-Ni_A_/C was lower than that on Pt-Ni_C_/C. These results show that the amorphous structure of Ni in Pt-Ni/C improves electrocatalytic capability for methanol oxidation over that of crystalline Ni in Pt-Ni/C.

**Figure 7:**
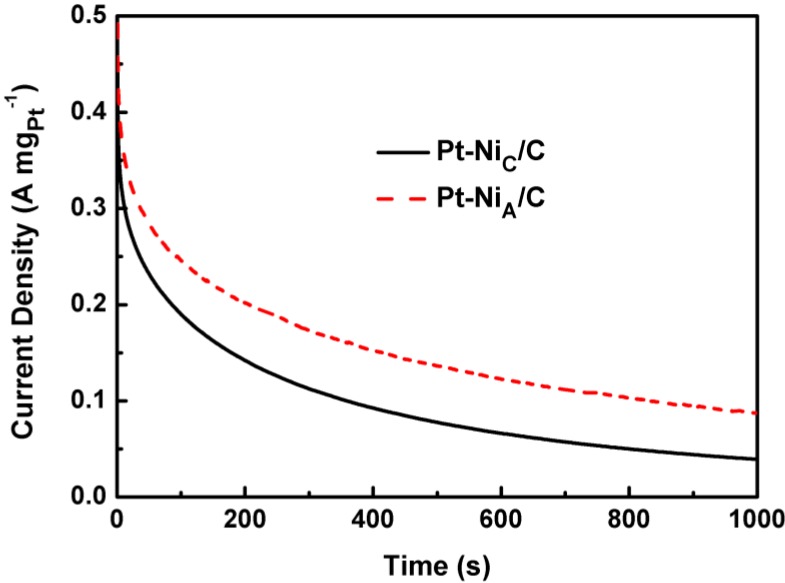
Chronoamperometric curves for methanol oxidation at 0.6 V *vs.* Ag/AgCl on Pt-Ni_A_/C and Pt-Ni_C_/C in 0.5 mol L^−1^ CH_3_OH + 0.5 mol L^−1^ H_2_SO_4_ solution at room temperature.

The superiority of the Pt-Ni_A_/C was further confirmed by chronoamperometry tests (Figure 7) recorded at a constant potential of 0.6 V at room temperature. The oxidation current decreased continuously for all the catalysts, most likely due to the formation of intermediate and poisoning species, such as CO_ads_, CH_3_OH_ads_, COOH_ads_, and CHO_ads_, during the methanol oxidation reaction [31]. The steady-state current density of Pt-Ni_A_/C was higher than that of the Pt-Ni_C_/C over the entire testing time, indicating that the Pt-Ni_A_/C catalyst had the best electrocatalytic activity towards the oxidation of methanol. The residual current densities of Pt-Ni_A_/C and Pt-Ni_C_/C after 1000 s were 0.087 A mg_Pt_^−1^ and 0.039 A mg_Pt_^−1^, respectively.

In summary, the electrochemical results showed that Pt-Ni_A_/C had higher methanol oxidation activity than Pt-Ni_C_/C. Due to the higher corrosion resistance of Ni_A_ than Ni_C_, a more uniform and thin Pt shell is expected to form on the surface of Ni_A_ nanoparticles, and hence result in the higher Pt-Ni interaction [32]. The stronger Pt-Ni interaction in Pt-Ni_A_/C likely also results in a decrease of adsorption energy of poisoning CO species allowing their efficient removal from the surface of Pt-Ni_A_/C [33].

## 3. Experimental Section

### 3.1. Preparation of Carbon-Supported Crystalline and Amorphous Ni nanoparticles

Ni_C_/C nanoparticles were synthesized using 304.0 mg nickel (II) chloride hexahydrate (NiCl_2_·6H_2_O) dissolved in a solution containing 25 mL ultrapure water and 25 mL ethanol, to which 413.4 mg of sodium citrate was then added with stirring. The solution was adjusted to pH 14 by addition of 5 wt % KOH/EG solution. Pretreated carbon black Vulcan XC-72R (300 mg) was added to the mixture under stirring, followed by addition of 10 mL of hydrazine hydrate. The mixture was transferred to a Teflon-lined autoclave container and heated at 120 °C for 4 h. The final product was collected by filtration, washed with ultrapure water (until the filtrate wash water reached pH 7), and finally dried under vacuum at 323 K for 12 h.

Ni_A_/C nanoparticles were prepared as reported previously [14]. Here, 413.4 mg sodium citrate was added to 35 mL of ethylene glycol (EG) and stirred for 0.5 h. Then 304.0 mg Nickel (II) chloride hexahydrate (NiCl_2_·6H_2_O) was added to the above solution and stirred for 20 min to completely dissolve the mixture. The pH value of solution was adjusted to 9~10 by addition of a 5 wt % KOH/EG solution with stirring. Pretreated carbon black Vulcan XC-72R (300 mg) was added and the solution was mixed in a flask using ultrasonic stirring, followed by heating at 433 K for 20 h. The final product was cooled, collected by filtration and washed with ultrapure water until the wash water read pH 7. The Ni_A_/C nanoparticles were finally dried at 323 K for 12 h under vacuum.

### 3.2. Preparation of Pt-Ni_C_/C and Pt-Ni_A_/C Catalysts

Pt shell deposition onto the surface of Ni/C nanoparticles was carried out as follows: 3.32 mL of20 mg mL^−1^ chloroplatinic acid (H_2_PtCl_6_·6H_2_O) aqueous solution and 30 mL ethylene glycol (EG) were added to a flask, and the pH value was adjusted to 9~10 using 5 wt % KOH/EG solution. Then, 100 mg of either Ni_C_/C or Ni_A_/C powder was added to the flask and the mixture was stirred for 6 h at 433 K. The final products were cooled and collected by filtration, washed with ultrapure water, and dried under vacuum at 323 K for 12 h, the obtained products were denoted as Pt-Ni_C_/C and Pt-Ni_A_/C, respectively.

### 3.3. Characterization

X-ray diffraction (XRD) patterns of the catalysts were recorded on a Shimadzu XD-3A (Japan), using filtered Cu-Kα radiation. Transmission electron microscopy (TEM) measurements were carried out on a JEM-2010 Electron Microscope (Japan) with the acceleration voltage of 200 kV. X-ray photoelectron spectroscopy (XPS) (Phi-5702 America) with a monochromatic Al Kα X-ray source (*hν* =1486.6 eV) was used. The chemical composition of the samples was determined by an IRIS advantage inductively coupled plasma atomic emission spectroscopy (ICP-AES) system (Thermo, America) and energy dispersive X-ray analysis (EDX) coupled to TEM.

Electrochemical measurements were carried out on a CHI650d electrochemical work station. A common three-electrode electrochemical cell was used, in which the counter and reference electrodes were a platinum wire and an Ag/AgCl (3 mol L^−1^ KCl) electrode, respectively. The working electrode *i.e.*, the catalyst-film electrode, was prepared as follows: 5 mg of catalyst was dispersed ultrasonically in 1.00 mL Nafion/ethanol (0.25% Nafion) for 15 min. 8 μL of the dispersion was transferred onto the glassy carbon disk (5 mm in diameter) using a pipette and then dried in the air. Then, the working electrode was prepared. The catalysts were characterized by cyclic voltammetry (CV) and chronoamperometry (CA) tests at room-temperature. Before each measurement, the solution was purged with high purity N_2_ for at least 10 min to ensure O_2_ free measurements. All current densities reported in this article were normalized to the electrode surface area (*S_EAS_*).

## 4. Conclusions

Carbon-supported Pt-Ni/C catalysts with either amorphous or crystalline Ni cores were synthesized and characterized. The XRD results showed that neither amorphous nor crystalline Ni structure as a seed crystal affected the structure of the Pt shell. However, the Pt-Ni interaction was different, as shown by the shift of Pt 4f XPS. Electrochemical results indicate that Pt-Ni_A_/C has greater methanol oxidation activity than Pt-Ni_C_/C, mainly because of the decrease in adsorption energy toward CO species owing to the stronger Pt-Ni interaction in the Pt-Ni_A_/C catalyst. Thus, controlling the crystallinity of the core metal affords a promising approach to improve the catalytic activity of core-shell-structure catalysts.
